# Clinical outcomes after online adaptive MR-guided stereotactic body radiotherapy for pancreatic tumors on a 1.5 T MR-linac

**DOI:** 10.3389/fonc.2023.1040673

**Published:** 2023-10-03

**Authors:** Hidde Eijkelenkamp, Guus Grimbergen, Lois A. Daamen, Hanne D. Heerkens, Saskia van de Ven, Stella Mook, Gert J. Meijer, Izaak Q. Molenaar, Hjalmar C. van Santvoort, Eric Paulson, Beth Ann Erickson, Helena M. Verkooijen, William Adrian Hall, Martijn P. W. Intven

**Affiliations:** ^1^ Department of Radiotherapy, University Medical Center Utrecht, Utrecht, Netherlands; ^2^ Department of Radiotherapy, Radboud University Medical Center, Nijmegen, Netherlands; ^3^ Department of Surgery, Regional Academic Cancer Center Utrecht, Utrecht, Netherlands; ^4^ Department of Radiation Oncology, Medical College of Wisconsin, Milwaukee, WI, United States; ^5^ Division of Imaging, University Medical Center Utrecht, Utrecht, Netherlands

**Keywords:** MRgRT, clinical outcomes, quality of life, toxicity, pancreatic cancer

## Abstract

**Introduction:**

Online adaptive magnetic resonance-guided radiotherapy (MRgRT) is a promising treatment modality for pancreatic cancer and is being employed by an increasing number of centers worldwide. However, clinical outcomes have only been reported on a small scale, often from single institutes and in the context of clinical trials, in which strict patient selection might limit generalizability of outcomes. This study presents clinical outcomes of a large, international cohort of patients with (peri)pancreatic tumors treated with online adaptive MRgRT.

**Methods:**

We evaluated clinical outcomes and treatment details of patients with (peri)pancreatic tumors treated on a 1.5 Tesla (T) MR-linac in two large-volume treatment centers participating in the prospective MOMENTUM cohort (NCT04075305). Treatments were evaluated through schematics, dosage, delivery strategies, and success rates. Acute toxicity was assessed until 3 months after MRgRT started, and late toxicity from 3–12 months of follow-up (FU). The EORTC QLQ-C30 questionnaire was used to evaluate the quality of life (QoL) at baseline and 3 months of FU. Furthermore, we used the Kaplan–Meier analysis to calculate the cumulative overall survival.

**Results:**

A total of 80 patients were assessed with a median FU of 8 months (range 1–39 months). There were 34 patients who had an unresectable primary tumor or were medically inoperable, 29 who had an isolated local recurrence, and 17 who had an oligometastasis. A total of 357 of the 358 fractions from all hypofractionated schemes were delivered as planned. Grade 3–4 acute toxicity occurred in 3 of 59 patients (5%) with hypofractionated MRgRT and grade 3–4 late toxicity in 5 of 41 patients (12%). Six patients died within 3 months after MRgRT; in one of these patients, RT attribution could not be ruled out as cause of death. The QLQ-C30 global health status remained stable from baseline to 3 months FU (70.5 at baseline, median change of +2.7 [P = 0.5]). The 1-year cumulative overall survival for the entire cohort was 67%, and that for the primary tumor group was 66%.

**Conclusion:**

Online adaptive MRgRT for (peri)pancreatic tumors on a 1.5 T MR-Linac could be delivered as planned, with low numbers of missed fractions. In addition, treatments were associated with limited grade 3–4 toxicity and a stable QoL at 3 months of FU.

## Introduction

Pancreatic cancer (PC) is often considered a systemic disease with a high risk of metastases and poor overall survival (1–3). Despite the systemic nature of the disease, local treatment remains essential for the survival and quality of life (QoL) of this patient group (4–7). Surgery has historically been considered as the only local treatment that can offer curative intent. With current advances in radiotherapy, however, radiotherapy has also become another effective option for patients with PC (8). In some series, the clinical outcomes of patients treated with high-dose radiation approach that of surgery (9).

It is complicated to irradiate PC to a high dose capable of eradicating the tumor. The anatomy in the pancreatic region changes considerably throughout the day and with every breath. Beyond local tumor movement, there is difficulty with daily tumor localization, discrimination between the tumor and radiosensitive local organs at risk (OAR), and the possibility to adjust the radiotherapy treatment plan accordingly. Magnetic resonance-guided radiotherapy (MRgRT) may allow for high-precision treatment intensification with the aim of improving clinical outcomes without increasing radiation-induced toxicity (6, 10–13). By visualizing the tumor and OAR at every fraction and minutes before dose delivery, the treatment plan can be improved by adapting it to the daily anatomy (14, 15). Visualization and daily adaptation of the treatment plan are especially relevant for mobile and centrally located tumors such as in the pancreatic region.

In recent years, MRgRT has been introduced in a growing number of centers worldwide and is increasingly being used to treat tumors in the upper abdomen. However, clinical outcomes have only been reported on a small scale, often retrospective, by single institutes, or in the context of clinical trials, in which patient selection might limit generalization of findings (6, 11–13). The 1.5 Tesla (T) MR-linac (Unity, Elekta AB, SE) is an MRgRT platform that received CE marking in 2018 and was introduced in multiple clinics around the globe. At the same time, a prospective cohort study was initiated in which all cancer patients treated on the 1.5 T MR-linac were included to collect clinical and technical data, and patient-reported outcomes (NCT04075305). Prior results of the first 943 patients enrolled in the study as a whole have been published previously (16). The current study aimed to evaluate clinical outcomes of the first group of patients with pancreatic and peripancreatic tumors treated with online adaptive MRgRT on a 1.5 T MR-linac.

## Materials and methods

Patients with (peri-)pancreatic tumors treated with the 1.5 T MR-linac at Froedtert and Medical College of Wisconsin (MCW) and University Medical Center Utrecht (UMCU) were identified through the Multi-OutcoMe EvaluatioN of radiation Therapy Using the MR-linac Study (MOMENTUM) (NCT04075305), a stage 2b study according to the R-IDEAL framework (17, 18). We included patients with (1) primary pancreatic, ampullary, or cholangiocarcinoma; (2) isolated local recurrence of these primary tumor types; or (3) oligometastases at the pancreatic site. We excluded patients receiving surgery within 3 months after MRgRT to not confound the outcomes of RT. The outcomes of patients were censored after surgery if they were operated after 3 months of follow-up (FU). All patients received online adaptive MRgRT on a 1.5 T MR-linac between April 2019 and June 2022, from the moment the MR-linac was first operational for (peri)pancreatic tumors until the last data update in July 2022. Furthermore, all patients consented with MOMENTUM for prospective collection of their clinical and technical data and patient-reported outcomes.

### Treatment

Patients were treated according to local treatment protocols. Both institutes used similar workflows and dose constraints (19, 20). The gross tumor volume (GTV) was delineated on the mid-position of a 3D T2w MR scan. MCW used separated clinical target volumes (CTV) for irradiating regional lymph nodes. These CTVs typically included the superior mesenteric artery (SMA), celiac artery (CA), and superior mesenteric vein (SMV) with modified margins ranging from 1 to 3 cm. In addition, the regions around the primary tumor that felt at risk for local tumor extension and spread were included. UMCU did not use CTVs. The planned target volume (PTV) was calculated as the GTV or CTV with an isotropic 3–5-mm margin. Dose prescription for coverage aimed for at least 95% of the PTV to receive 99% of the dose and at least 100% of the GTV to receive 99% of the dose. Coverage was reduced if dose constraints on organs at risk were compromised. The radiation oncologists at both centers used an adapt-to-position (ATP) or adapt-to-shape (ATS) strategy to adjust for the daily anatomy during the planning of each fraction (21). In short: with ATP, the pretreatment plan is moved rigidly to align with the new anatomical situation. With ATS, all delineations are manually adapted to their new position and shape, after which a new treatment plan is calculated. In addition, the biologically effective dose (BED) was calculated, with a tumor α/β ratio of 10, from the prescription dose to compare fractionation schemes (22). The standard fractionation scheme was a five-fraction regimen. A conventional fractionation scheme was chosen for patients with concurrent chemotherapy or other medical indication. Furthermore, UMCU used a customized abdominal corset for motion reduction (23).

The oncological treatment history per patient was registered up to 5 years prior to MRgRT. Only oncological treatments given directly prior to MRgRT were summarized in the treatment overview.

### Data collection

Patient characteristics and treatment details were registered in patient files. Clinical Research Coordinators from the MOMENTUM study collected the data from these files and entered them into the MOMENTUM database. Technical data, such as imaging data, were collected automatically and transferred to a technical study database.

### Toxicity reporting

Acute toxicity was defined as symptoms experienced from start MRgRT to 3 months FU, and late toxicity between 3 and 12 months. The standard FU moments were during RT and around 1 week, 1 month, and 3 months after RT. The clinicians reported their patients’ toxicities/symptoms as defined by the Common Terminology Criteria for Adverse Events (CTCAE) version 5. After RT in the upper abdomen, the following toxicities were assessed standardly during FU and noted in the MOMENTUM registry: abdominal pain, anorexia, bloating, constipation, diarrhea, fatigue, gastritis, gastroparesis, malabsorption, nausea, pancreatitis, vomiting, and weight loss. In addition, clinicians could report other grade 3 or higher toxicities/symptoms that were observed during FU. The highest toxicity grade experienced between the FU moments was registered.

### Quality of life

Patients with pancreatic tumors within MOMENTUM are asked to fill in the EORTC QLQ-C30 and PAN26 questionnaires on QoL. They receive these questionnaires at baseline and at 3, 6, 12, and 24 months at home. From the QLQ-C30 questionnaire, we calculated global health status (GHS), as well as functional and symptom scales (range 0–100) according to the EORTC scoring manual (24). For the GHS and functional scales, a high score reflects a high QoL or good functioning, whereas for the symptom scales a high score indicates higher symptom severity.

### Outcomes

Patients with 3 months of FU were assessed for their early toxicity and QoL outcomes. In addition, MRI tumor response assessment was registered at 3 months of FU according to the RECIST criteria (25). Survival was measured from the start of MRgRT until the date of death or last FU for the whole cohort and stratified by tumor type: primary tumor, isolated local recurrence, and oligometastasis. The primary tumor group consisted of all patients with a primary borderline resectable tumor, a primary locally advanced tumor, or medically inoperable patients with primary tumors.

### Statistical analysis

Patient, tumor, and treatment characteristics and toxicity were assessed for each patient individually and presented as median with range or interquartile range (IQR), mean with standard deviation (SD), or frequency with percentage, depending on their distribution. QoL at 3 months of FU was compared with baseline QoL by calculating a paired-samples T test in patients who filled in questionnaires at both time points. The independent-samples T test was used for cross-sectional analysis at baseline to determine if patients who did fill in the 3-month FU questionnaire had significantly different QoL scores than those who did not. A Kaplan–Meier curve analysis was performed to assess cumulative overall survival.

Analyses were performed using Statistical Package for Social Sciences (SPSS) version 25 (Released 2017. IBM SPSS Statistics for Windows, Version 25.0. Armonk, NY: IBM Corp.).

## Results

### Patient and treatment characteristics

Patient characteristics and treatment details are shown in **Table 1** (tumor details in **Supplementary 1**). There were 11 patients that were excluded who had surgery within 3 months of follow-up. A total of 80 patients with a median age of 67 years were included. The median FU time was 8 months with a range from 1 to 39 months. There were 34 of 80 patients (43%) who had a primary tumor; of these tumors, nine were assessed as resectable. Another 29 patients (36%) had an isolated local recurrence, and 17 patients (21%) had an oligometastasis in the peripancreatic region. There were 23 of 80 patients (29%) who received a form of oncological treatment, up to 3 months prior to MRgRT. All of them received chemotherapy (N = 23), of whom one also received immunotherapy. During the course of MRgRT, 13 of 80 patients (16%) received concurrent chemotherapy, of whom one also received immunotherapy. Furthermore, four patients underwent surgery between 3 and 6 months of FU.

**Table 1:** Baseline characteristics and treatment details.

Baseline characteristics	N=80	Treatment details	N=80
Sex	N (%)	Dose plan	Median (range)
Male	41 (51)	Total dose	40 Gy (32-70)
Female	39 (49)	Number of fractions	5 (5-31)
Age	Years	GTV	19 cc (1-142)
Median	67	PTV	84 cc (5-466)
Range	38 - 90	Treatment schemes	N (%)
Institute	N (%)	5 x 6.6 Gy	4 (5)
UMCU	57 (71)	5 x 7.0 Gy	10 (13)
MCW	23 (29)	5 x 8.0 Gy	56 (70)
Tumor type	N (%)	8 x 5.0 Gy	1 (1)
Primary	34 (43)	31 x 2.25 Gy	3 (4)
Isolated local recurrence	29 (36)	Other conventionally fractionated	
Oligometastasis	17 (21)	schemes	6 (8)
KPSS	N=38	Treatment completion	N (%)
Median	90%	Entire doseplan received	77 (96)
IQR	80-100%	Missed 1 Fx	1 (1)
ECOG performance status	N=46	Unknown	2 (3)
Median	1	Online adaptive strategy	N (%)
IQR	0-1	Hypofractionated schemes	
CA 19-9	N=41	ATS	69 (86)
Median	82 U/mL	ATP	1 (1)
IQR	22-711 U/mL	Mixed*	1 (1)
Other oncological therapy	N (%)	Conventionally fractionated schemes	
Before radiotherapy*	23 (29)	ATP	8 (10)
hemotherapy	23	Mixed**	1 (1)
Hormone therapy	1		
Concurrent with radiotherapy	13 (16)		
Chemotherapy	13		
Immunotherapy	1		

UMCU, University Medical Center Utrecht; MCW, Froedtert and Medical College of Wisconsin; IQR, interquartile range; Gy, gray; GTV, gross tumor volume; PTV, planned target volume; Fx, fractions; ATP, adapt-to-position; ATS, adapt-to-shape. *Other oncological therapy given directly before start radiotherapy. **Mixed was a ATS/ATP ratio.

In total, 71 of 80 patients (89%) received a hypofractionated RT scheme, with 357 of the 358 fractions being delivered as planned. The remaining fraction was canceled due to disease exacerbation, and one treatment had a mixed online strategy with a 4:1 ATS/ATP ratio (20). In addition, 9 of 80 patients (11%) were treated with conventionally fractionated schemes (10–31 fractions), of which one treatment had a mixed online strategy with a 23:7 ATS/ATP ratio. Two patients did not complete their conventional scheme: one patient had 20 fractions planned but missed an unknown number of fractions due to disease exacerbation, and the reason and number of missed fractions were unknown for the remaining patient with 63 Gy in the 21-fraction planned treatment. In total, 63 of 80 patients (79%) received at least a planned BED of 72 Gy.

### Toxicity

Acute and late toxicities were assessed in 66 and 46 patients, respectively, of whom 28 completed at least 12 months of FU. Two patients experienced both acute and late grade 3 toxicity. In addition, two patients had preexisting grade 3 symptoms at 3 months of FU; one had abdominal pain, and one had decreased lymphocyte count, and both were not considered radiotherapy-related toxicity. An overview of the four most frequently reported toxicities at 12 months of FU is illustrated in **Table 2**. An overview of the remaining clinician-reported toxicities is illustrated in **Supplementary 2**. The toxicities described below are grade 3 unless indicated otherwise.

**Table 2:** The four most frequent reported symptoms at baseline, at 3, 6, and 12 months of follow-up with grading according to the CTCAE v5. No grade 4 of these symptoms was observed.

		Baseline (N=80)	3 MFU (N=66)	6 MFU (N=46)	12 MFU (N=28)
Abdominal pain					
Grade 0-2		78 (98%)	65 (98%)	46 (100%)	25 (89%)
Grade 3		2 (2%)	1 (2%)	0 (0%)	3 (11%)
Diarrhea					
Grade 0-2		80 (100%)	66 (100%)	46 (100%)	28 (100%)
Grade 3		0 (0%)	0 (0%)	0 (0%)	0 (0%)
Fatigue					
Grade 0-2		80 (100%)	66 (100%)	45 (98%)	27 (96%)
Grade 3		0 (0%)	0 (0%)	1 (2%)	1 (4%)
Nausea					
Grade 0-2		80 (100%)	66 (100%)	46 (100%)	28 (100%)
Grade 3		0 (0%)	0 (0%)	0 (0%)	0 (0%)

M, month; FU, follow-up.

Acute grade 3–4 toxicity occurred in 5 of 66 patients (8%). For patients with hypofractionated schemes, this toxicity occurred in 3 of 59 patients (5%). One patient with distant progressive disease had anemia. One patient had anemia and tumor-related portal hypertension on MR imaging. One patient had biliary obstruction by a new (outfield) regional metastasis. One patient with concurrent chemotherapy had multiple grade 3 toxicities—atrial flutter, dehydration, dizziness, peripheral vascular disease, and urinary tract infection—and grade 4 paroxysmal atrial tachycardia. The last patient with concurrent chemotherapy and stable disease on imaging had anorexia, malabsorption, and gastric hemorrhage.

Late grade 3–4 toxicity occurred in 7 of 46 patients (15%). For patients with hypofractionated schemes, this toxicity occurred in 5 of 41 patients (12%). Two patients received subsequent chemotherapy, of which one patient with regional progressive disease experienced fatigue at 6 months of FU and one patient with stable disease on imaging had ascites and a cough at 6 months of FU and ascites, anemia, fatigue, lung infection, and peritoneal infection at 12 months of FU. One patient with new bone metastases had a lung infection at 6 months FU. At 12 months of FU, this patient had developed a new liver metastasis and experienced gastric obstruction, hypotension, lung infection, pancreatitis, and grade 4 paroxysmal atrial tachycardia. Two patients with new liver metastases experienced multiple toxicities; one had a hepatic hemorrhage and abdominal pain at 12 months of FU, and one had abdominal pain, anorexia, duodenal ulcer, sepsis, syncope, and urinary tract infection at 12 months of FU. One patient with a partial response on imaging had abdominal pain at 12 months of FU. The last patient with stable disease on imaging had portal vein thrombosis and ascites at 6 months of FU.

### Quality of life

The GHS and the functional and symptom scales from the EORTC QLQ-C30 questionnaire are shown in **Supplementary 3**. The QLQ-C30 had a response rate of 63% (N = 50 of 80) at baseline and 42% (N = 28 of 66) at 3 months of FU. The GHS remained stable from baseline to 3 months of FU (70.5 at baseline, median change of +2.7 [P = 0.5]). No significant differences were seen in the functioning and symptom scales, as shown in **Table 3**. Cross-sectional analysis at baseline showed no significant differences in scores between patients who did (n = 28) or did not (n = 22) have a filled-in questionnaire at 3 months of FU (**Supplementary 4**).

**Table 3:** Analysis of 28 patients who filled in both baseline and 3-month follow-up QLQ-C30 questionnaires.

QLQ-C30	Baseline	SD	FU 3M	SD		
(N=28)	Mean		Mean			P-value
Global health status						
Global health status	70.5	19.3	73.2	15.3		0.452
Functional scales						
Physical functioning	81.9	21.9	78.6	20.7		0.316
Role functioning	77.4	28.4	75.0	25.9		0.667
Emotional functioning	80.4	20.2	78.9	18.4		0.617
Cognitive functioning	85.1	22.4	88.1	18.6		0.379
Social functioning	80.4	26.5	84.5	19.7		0.409
Symptom scales / items						
Fatigue	29.8	24.6	31.7	26.3		0.716
Nausea and vomiting	10.7	19.4	9.5	14.6		0.752
Pain	19.0	22.6	22.6	26.9		0.463
Dyspnea	9.9	18.1	14.8	21.4		0.212
Insomnia	23.8	27.0	28.6	28.3		0.161
Appetite loss	11.9	22.6	15.5	23.1		0.541
Constipation	10.7	18.3	10.7	15.9		1.000
Diarrhea	14.3	29.3	20.2	22.8		0.306
Financial difficulties	7.1	13.9	7.1	13.9		1.000

Scores are from 0 to 100 calculated with the EORTC QLQ-C30 scoring manual (22). For the global health status and functional scales, a high score reflects a high quality of life or good functioning, whereas a high score indicates higher symptom severity for the symptom scales. FU 3M, follow-up 3 months; SD, standard deviation.

The QLQ-PAN26 questionnaire was completed both at baseline and 3 months of FU by 23 of 66 patients (35%). None of the items significantly differed between these time points (**Supplementary 5**).

### Treatment response

Of 66 patients who completed 3 months FU, two patients had a complete response (3%), 10 patients had a partial response (15%), and 23 patients had RECIST stable disease (35%). Disease progression occurred in 14 patients (21%), of whom four had local progression, one had regional progression, eight had distant progression, and one had both local and distant progression. Treatment response was unknown in 17 patients because FU imaging was unavailable.

### Survival

**Figure 1:**
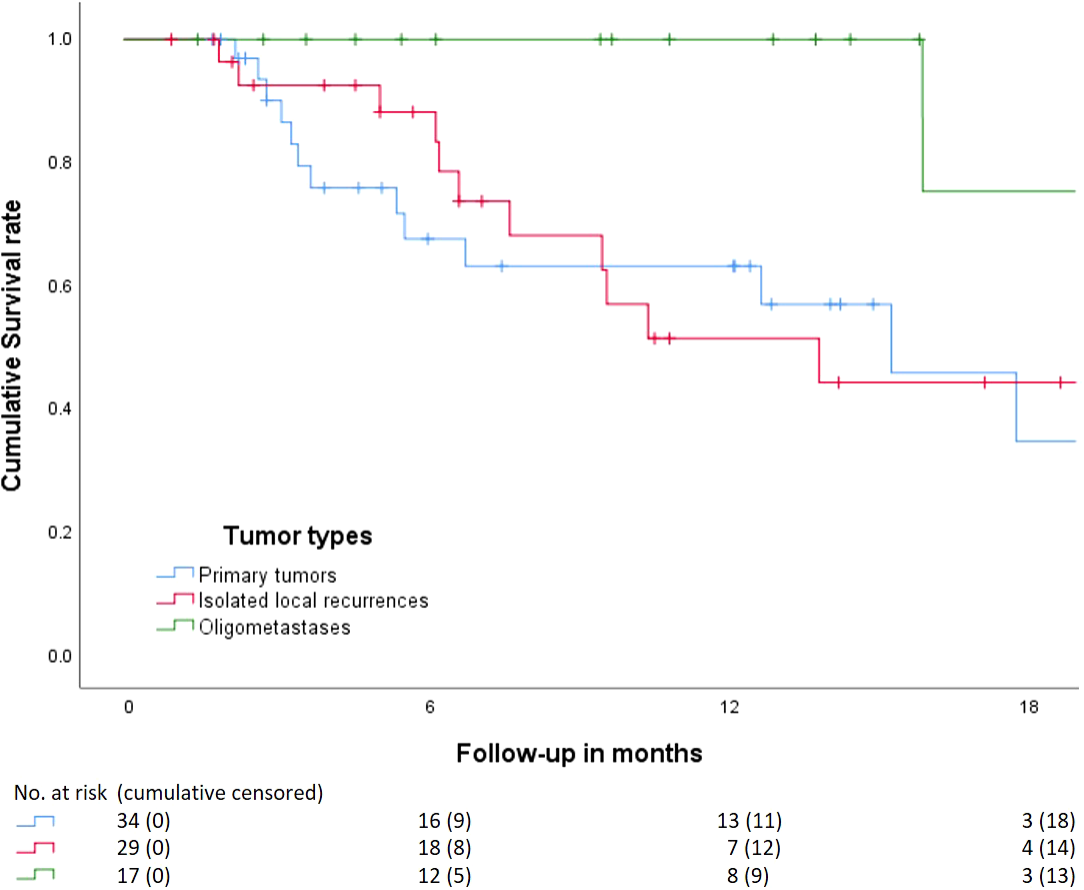
Kaplan–Meier curve analysis on the cumulative overall survival from start MRgRT stratified by tumor type. Primary tumors, isolated local recurrence, and oligometastasis. Beneath the numbers at risk, and patients censored cumulatively. Four patients with primary tumors received surgery between 3 and 6 months after which they were censored.

For the entire cohort (N = 80), 6-month and 1-year survivals were 83 and 67%, respectively (**Supplementary 6**). There were 19 patients who died within the first 12 months of FU. After stratification for tumor type, 6-month and 1-year survival for primary tumors (N = 34), isolated local recurrences (N = 29), and oligometastases (N = 17) were 70% and 66%; 88% and 51%, and 100% and 100%, respectively (**Figure 1**). Six patients died within 3 months after treatment. One patient treated for an isolated local recurrence of PC had progressive peritoneal disease at 1 month of FU and died after best-supportive care. One patient with locally advanced pancreatic cancer (LAPC), intestinal ingrowth, and tumor progression under FOLFIRINOX died 2 months after MRgRT. One patient with an isolated local recurrence of PC and gastrointestinal tumor ingrowth died of sepsis due to a perforation, in which RT attribution could not be ruled out. One inoperable patient with PC and a history of poor diabetes compliance died due to the consequences of hyperglycemia. One patient with LAPC and several comorbidities died due to a sudden out-of-hospital cardiac arrest. The last patient had PC with distant metastases prior to MRgRT and died due to systemic disease progression.

## Discussion

This study showed that online adaptive MRgRT on a 1.5 MR-linac in patients with (peri)pancreatic tumors could be delivered as planned in both centers, with only one missed treatment fraction in the hypofractionation group. In addition, treatment at the pancreatic area was associated with limited grade 3–4 toxicity and a stable QoL at 3 months of FU.

This study reported outcomes of the 1.5 T MR-linac for inoperable PC, using a relatively large cohort of patients treated in two international centers. We observed a low incidence of toxicity attributable to radiation, which encourages the feasibility of using this technology to apply dose intensification with online adaptive MRgRT in the pancreatic area.

MRgRT is an upcoming treatment modality introduced into clinical practice recently but has shown promise for further reducing radiotoxicity compared with conventional techniques or cone-beam computed tomography (CBCT) guidance. A study by Tchelebi et al. summarized that conventionally fractionated chemoradiotherapy (50.4 Gy–60 Gy) has around 40% grade 3–4 acute toxicity for locally advanced pancreatic cancer (10). CBCT-guided stereotactic RT has shown lower rates of grade ≥3 toxicity to 6%–10% with total doses of 30 Gy–40 Gy in five fractions (26–28). In contrast, studies using a 0.35-T MRIdian observed 0%–3% grade 3–4 acute toxicity and 3%–5% late toxicity with prescribed BED ranging from 72 Gy to 100 Gy for inoperable PC (6, 11, 12). A recent study using a 1.5 T MR-linac found no grade 3–4 acute and late toxicity with 100-Gy BED prescribed to inoperable pancreatic tumors (summary in **Supplementary 7**) (13). In line with these studies, we observed minimal grade 3–4 toxicity in the hypofractionation group after including all experienced symptoms, except preexisting. Acute toxicity grades 3–4 occurred in only 3 of 59 patients (5%) and late toxicity in 5 of 41 patients (12%). Six patients died within 3 months after MRgRT, of which in one cause of death, RT attribution could not be ruled out. This patient had gastrointestinal tumor ingrowth before RT started and died of sepsis due to gastrointestinal perforation. These mortality rates are comparable with literature (28).

To our knowledge, this is the first MRgRT study for PC to report on QoL. Our QoL data have shown stable QLQ-C30 and QLQ-PAN26 scores with no significant differences at 3 months of FU compared with baseline. These outcomes are in line with CBCT-guided RT studies for PC. Herman et al. reported a lower GHS at baseline, which remained stable 4 months after RT (n = 22). However, they found a significant decrease in pancreatic pain with a median change of −8 at 4 weeks of FU (n = 43) (27). Reyngold et al. reported similar stable scores with a GHS of 72 and functional scales ranging from 77 to 86 (n = 18) at 3 months of FU (29). Furthermore, a large cohort study containing 629 pancreatic or periampullary cancer patients treated with different therapies showed, except for insomnia, lower overall health-related QoL scores in the intervention group compared with the scores in our cohort at 3 months of FU (30).

Rudra et al. reported a 1-year OS of 83% with high-dose SBRT (BED >70 Gy) on an MRIdian for 24 patients with inoperable PC (6). In some of these cases, the prescribed doses were higher with BED ranging to almost 98 Gy. For their standard-dose SBRT (BED <70 Gy) group of 20 patients, the 1-year OS was 45%. Other studies on MRgRT reported a 1-year OS ranging from 59% to 68% for inoperable PC (11–13). Most of these patients received 50 Gy in five fractions. These findings were similar in our cohort with a 1-year OS of 70% for the group of patients with primary (peri)pancreatic tumors (N = 34), who received a median dose of 40 Gy in five fractions. Differences in 1-year OS might be explained by different cohort sizes, total radiation doses, or treatment modalities. These results are significantly limited by the small number of patients to have been followed long-term, along with the heterogeneous cohort of patients included in our study.

An interesting finding in our cohort was particularly poor outcomes associated with locally recurrent patients. These patients had a median overall survival of 14 months. This group represented a meaningful percentage of the total patients included in this analysis (36%). Local recurrence events are very common in pancreatic cancer when patients are managed with surgery first (31, 32). Characterizing the outcomes of patients with local-only recurrences is important. There are several potential reasons for poor outcomes under these circumstances such as difficulty in defining the extent of the local recurrence, altered regional anatomy, and finally more aggressive disease biology (33, 34). The UMCU started a randomized controlled trial on MRgRT versus standard of care alone for isolated local pancreatic ductal adenocarcinoma recurrence (NCT04881487).

Our single-arm study was not a clinical trial and, unfortunately, could not report local control rates, considering that some patients did not receive standardized imaging during later FU. FU imaging for PC depends on shared-decision making between patient and clinician and might only be desired in some PC scenarios. Therefore, a future multicenter study with a control group, standardized FU imaging protocol, and local control as a primary objective could be considered. Another limitation of our study is that we included different types of (peri)pancreatic tumors, which caused considerable heterogeneity. As a result, oncological outcomes could not be clearly related to a specific tumor morphology, such as pancreatic ductal adenocarcinoma. Nevertheless, overall treatment results showed a low incidence of high-grade radiotoxicity in all tumor types. In addition, FU outcomes were sometimes missing and we do not know if these patients were doing well or poorly. However, at least 35 of 66 patients (53%) did not show progressive disease and five (8%) had local progression at 3 months of FU. Moreover, although the median FU in our cohort was 8 months and ranged 1 to 39 months, study outcomes of MRgRT for PC are still very early. Therefore, longer FU is required to provide more definite conclusions on survival outcomes.

Definitive conclusions on the modality’s potential can only be made after further investigation of clinical outcomes in combination with more testing, refinement, and practice (8). Future perspectives might include improvement of delivery technologies, better autosegmentation models, workflow optimization, or use of functional imaging applications (35). Even though 63 of 80 patients (79%) received a BED of 72 Gy, Reyngold et al. suggested that a BED of at least 100 Gy is required to provide an ablative effect on these tumors (36). Considering the low incidence of radiotoxicity presented in our study, we believe that online adaptive MRgRT with a 1.5 T MR-linac might be a safe and effective RT modality for escalation to an ablative dose in (peri)pancreatic tumors. This might further increase treatment response and overall survival without losing QoL.

In conclusion, online adaptive MRgRT with a 1.5 T MR-linac is feasible for pancreatic and peri-pancreatic tumors with limited adverse treatment effects and a stable QoL. These results pave the way for new multicenter studies on dose-escalation strategies with MRgRT to improve treatment response and overall survival while simultaneously maintaining QoL for patients with pancreatic tumors.

## Data availability statement

Secured data but available by request to the principle investigator. Requests to access the datasets should be directed to HV, H.M.Verkooijen@umcutrecht.nl.

## Ethics statement

Ethical review and approval was not required for the study on human participants in accordance with the local legislation and institutional requirements. The patients/participants provided their written informed consent to participate in this study.

## Author contributions

All authors listed have made a substantial, direct, and intellectual contribution to the work, and approved it for publication.
