# Supplementary material for: Clinical outcomes after online adaptive MR-guided stereotactic body radiotherapy for pancreatic tumors on a 1.5 T MR-linac
Source: Front Oncol. 2023 Oct 3;13:1040673. doi: 10.3389/fonc.2023.1040673 (PMC10579578; doi:10.3389/fonc.2023.1040673)
Supplement: Supplementary file 1 [file DataSheet_1.docx]

# **Supplementary materials**


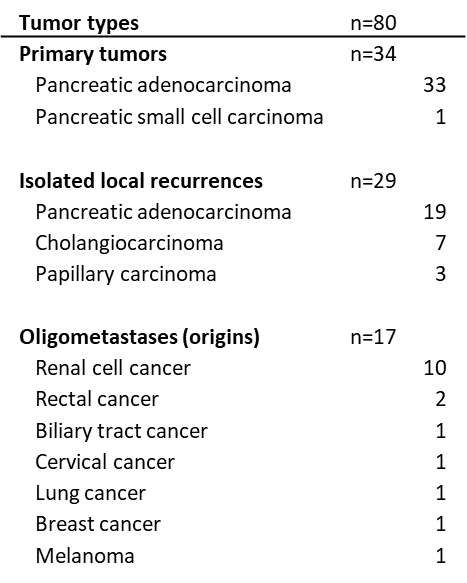


Suppl. 1: An overview of the treated tumor types in the cohort divided into three groups.

#
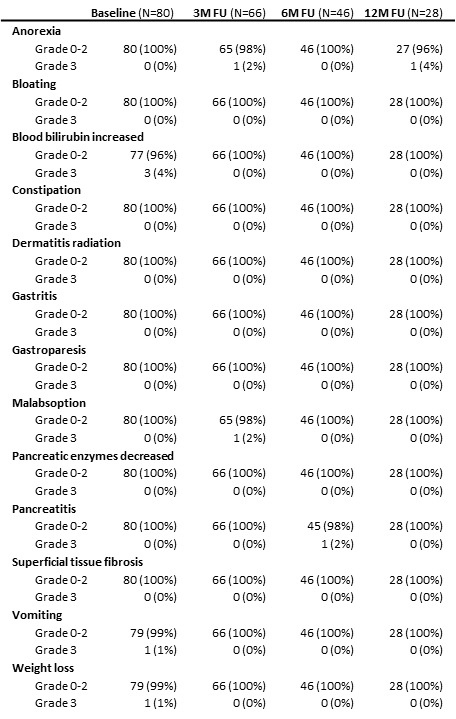


Suppl. 2: An overview of the remaining clinician reported toxicities with grading according to the CTCAE v5. No grade 4 of these symptoms was observed. Abbreviations: M, month; FU, follow-up.


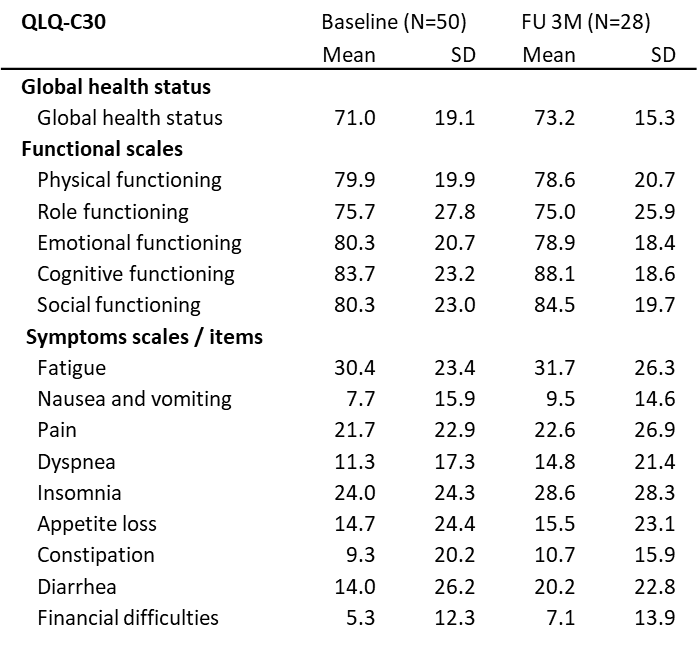


Suppl. 3: Results from the EORTC QLQ-C30 questionnaire on quality of life at baseline and three months follow-up. Scores are from 0 to 100 calculated with the EORTC QLQ-C30 scoring manual.^22^ For the global health status and functional scales, a high score reflects a high quality of life or good functioning, whereas a high score indicates higher symptom severity for the symptom scales. Abbreviations: FU 3M, follow-up at three months; SD, standard deviation.


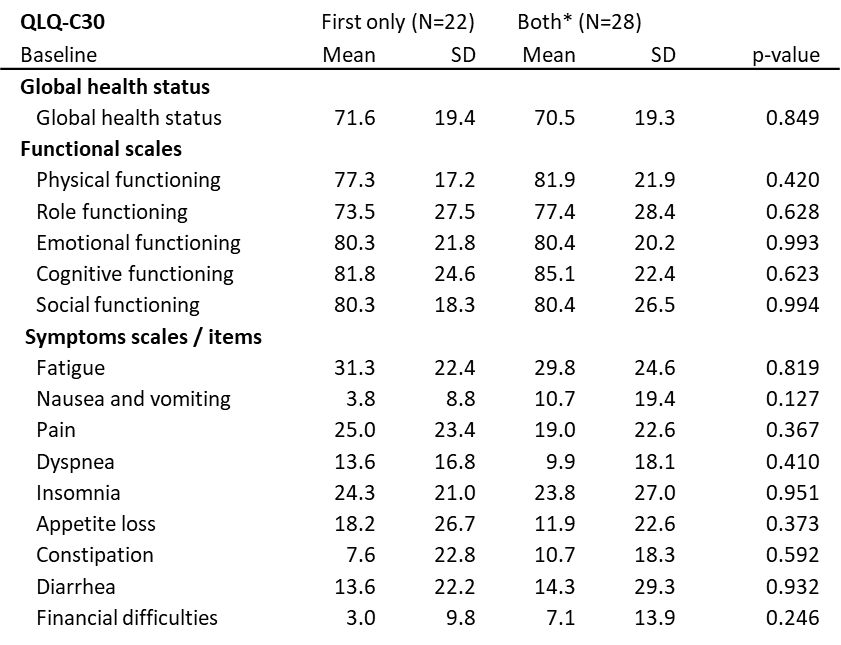


Suppl. 4: Cross-sectional analysis of the QLQ-C30 baseline results between patients who did (N=28 of 66) or did not (N=22 of 66) fill in the three months follow-up questionnaire along with the baseline questionnaire. *Both is the group who filled in the baseline as well as the three months follow-up questionnaire. Abbreviations: SD, standard deviation.


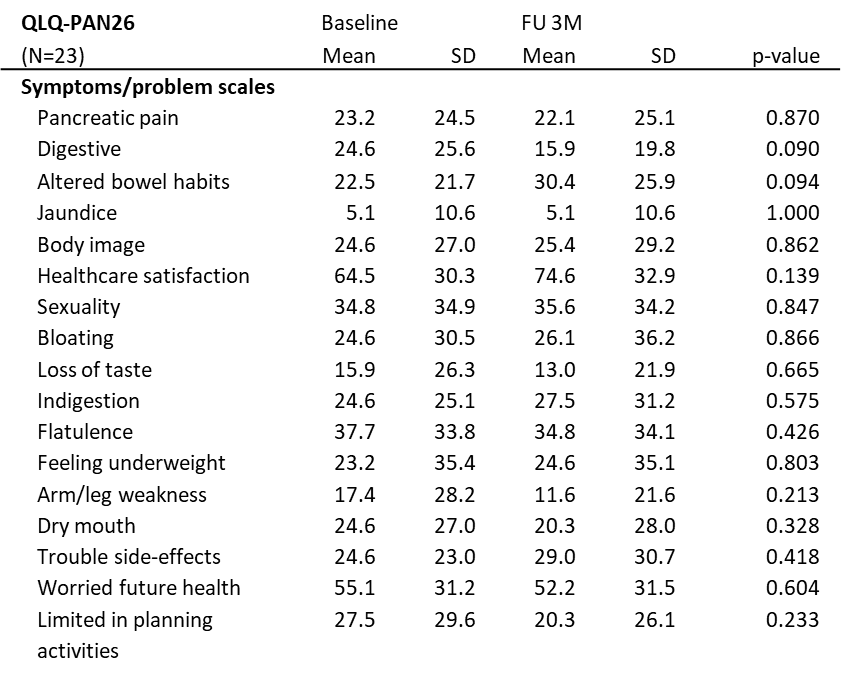


Suppl. 5: Analysis of 23 patients who filled in both baseline and three months follow-up EORTC QLQ-PAN26 questionnaires. Scores range from 0 to 100 with a high score indicates higher symptom/problem severity. Abbreviations: FU 3M, follow-up at three months; SD, standard deviation.


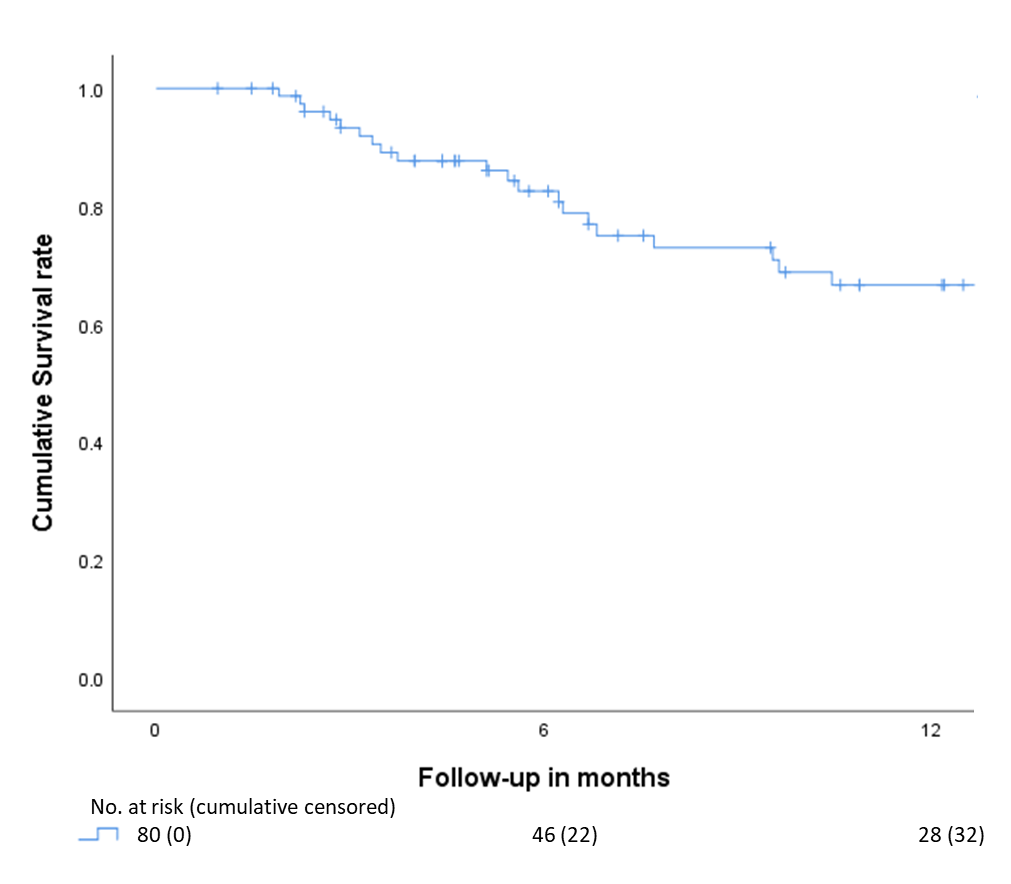


Suppl. 6: Kaplan-Meier curve analysis on cumulative overall survival of the whole cohort. Beneath the numbers at risk, and patients censored cumulatively.


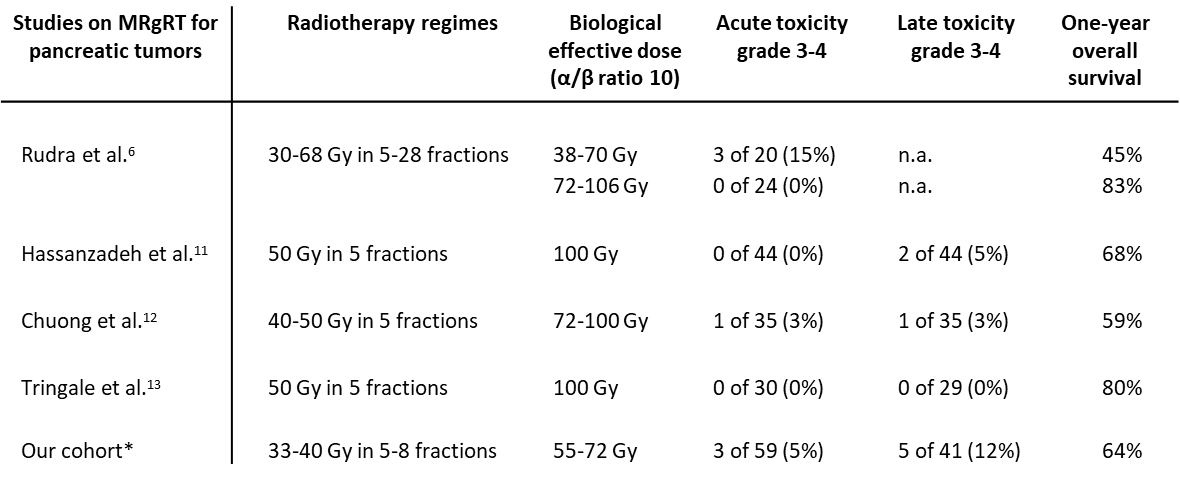


Suppl. 7: Summary table of studies using magnetic resonance guided radiotherapy for inoperable pancreatic tumors. Abbreviations: MRgRT, magnetic resonance guided radiotherapy; Gy, gray; n.a., not applicable. * This row represents the patients within this cohort who received a hypofractionation regime.
